# Supplementary material for: Agent-Based Modeling of T Cell Receptor Cooperativity
Source: Int J Mol Sci. 2020 Sep 4;21(18):6473. doi: 10.3390/ijms21186473 (PMC7555007; doi:10.3390/ijms21186473)
Supplement: Supplementary file 1 [file ijms-21-06473-s001.pdf]

Supplementary Materials

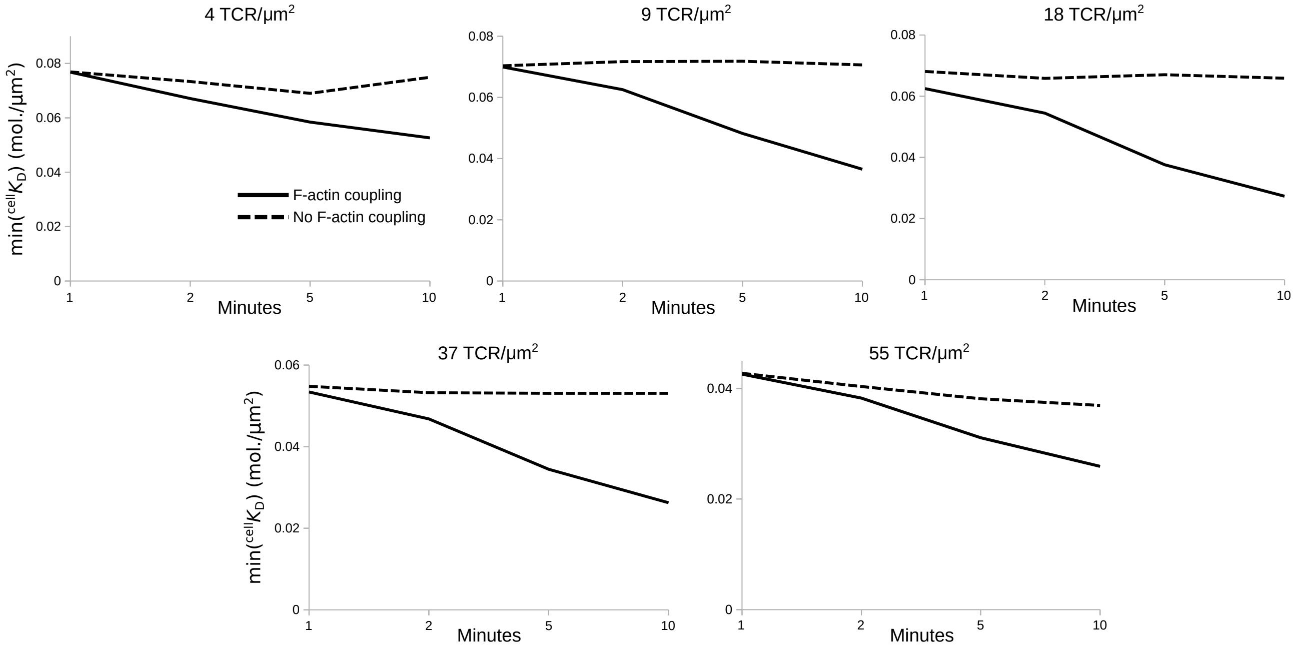

Figure 1: Time evolution of $\min({}^{\text{cell}}K_{\text{D}})$ with TCR and pMHC titration, in the presence (solid lines) or absence (dashed lines) of F-actin centripetal transport.

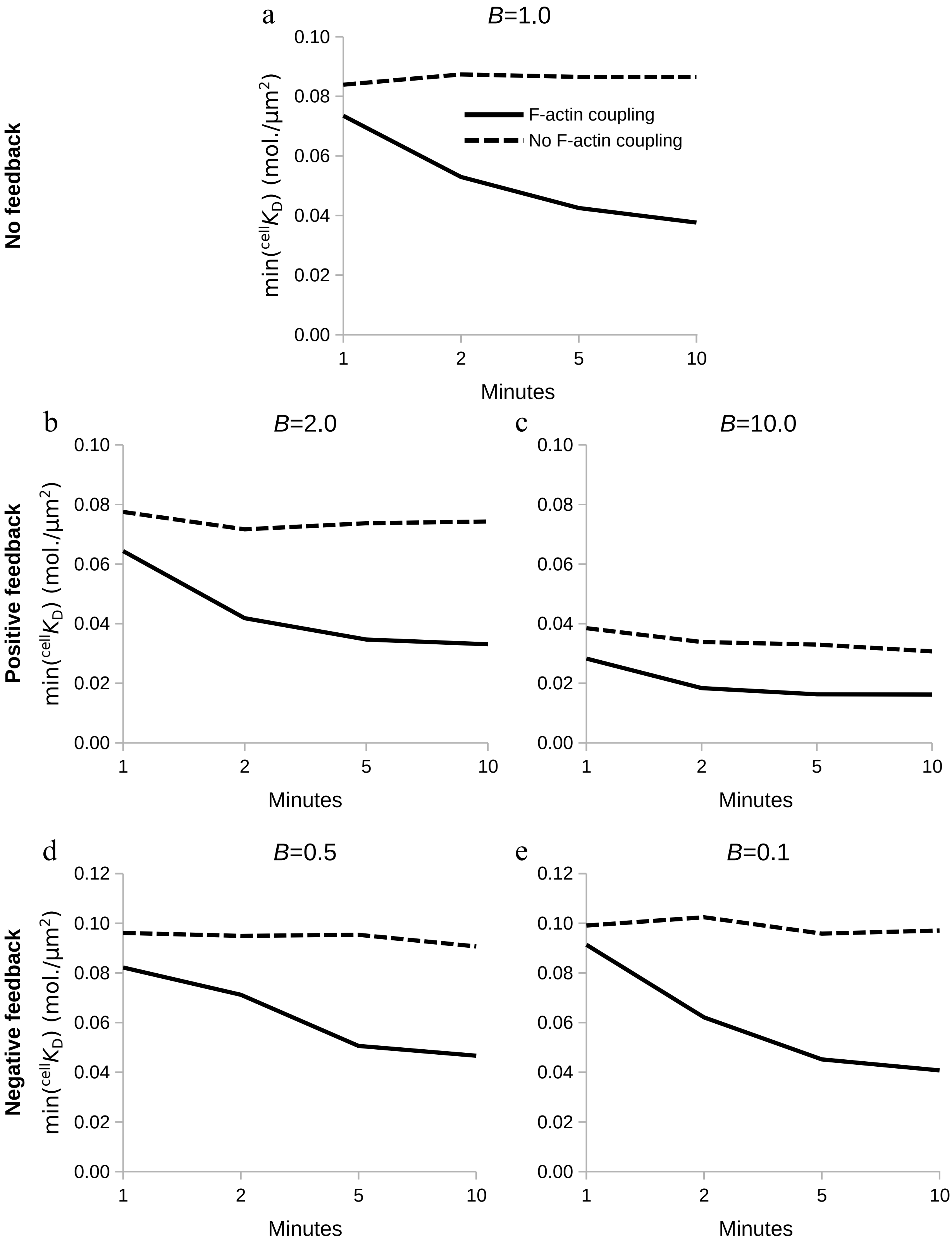

Figure 2: Time evolution of $\min({}^{\mathrm{cell}}K_{\mathrm{D}})$ with feedback from the binding coefficient, B, in the presence (solid lines) or absence (dashed lines) of F-actin centripetal transport.
